# Complete Mitochondrial Genome of *Apis cerana* (Hymenoptera: Apidae) from Two Geographical Regions: Insights into Structure and Genetic Differentiation

**DOI:** 10.3390/insects15120960

**Published:** 2024-12-02

**Authors:** Yuhui Chen, Runlang Su, Rui Zhu, Guiling Ding, Zhanbao Guo, Lin Du, Jiaxing Huang

**Affiliations:** 1 State Key Laboratory of Resource Insects, Institute of Apicultural Research, Chinese Academy of Agricultural Sciences, Beijing 100093, China; chenyuhui0902@163.com (Y.C.); surunlang@gmail.com (R.S.); 82101225470@caas.cn (R.Z.); dingguiling@caas.cn (G.D.); guozhanbao@caas.cn (Z.G.); 2 Key Laboratory for Insect-Pollinator Biology of the Ministry of Agriculture and Rural Affairs, Institute of Apicultural Research, Chinese Academy of Agricultural Sciences, Beijing 100093, China; 3 College of Animal Science and Technology, Yunnan Agricultural University, Kunming 650201, China; 4 Guizhou Livestock and Poultry Genetic Resources Management Station, Guiyang 550000, China; dulinbumblebee@sina.com

**Keywords:** honeybee, *Apis cerana*, mitochondrial genome, phylogenetic relationship, group differentiation

## Abstract

The honeybee *Apis cerana* is an important pollinator that is distributed across most regions in China. Mitochondrial DNA (mtDNA) is inherited maternally in bees; therefore, the mtDNA characteristics of a single bee can represent those of the entire colony. Comparing the mitochondrial genomes of *A. cerana* from different geographic regions via third-generation sequencing technology could provide insights into species differentiation and enable the identification of stable molecular markers to distinguish among geographically distinct populations. Here, we determined the complete mitochondrial genomes of the *Apis cerana*-Diannan and *Apis cerana*-Yun-Gui Plateau populations by using PacBio HiFi sequencing technology. The *A. cerana*-Diannan and *A. cerana*-Yun-Gui Plateau mitochondrial genome lengths were 16,214 and 16,304 bp, respectively. Both *A. cerana* mitochondrial genomes contained 13 protein-coding genes (PCGs), 22 transfer RNAs (tRNAs), 2 ribosomal RNAs (rRNAs) and an AT-rich region. The relative synonymous codon usage (RSCU) values for 13 PCGs were greater than 1. The length of the AT-rich region of *A. cerana* in different regions was different. Phylogenetic analyses revealed that these two geographically distinct *A. cerana* populations belong to the same phylogenetic branch, but the genetic distance between DN and YG is further than that between YG and *A. cerana*-Aba and *A. cerana*-Central China. Overall, *A. cerana* with different geographic distributions exhibit genetic diversity at the mitochondrial genome level. A large fragment was found to be inserted or deleted in the AT-rich region, shedding light on the genetic differentiation of *A. cerana* in different ecological environments.

## Simple Summary

Mitochondrial DNA is inherited maternally in bees, and the mitochondrial DNA characteristics of a single bee can represent those of the entire colony. We determined the complete mitochondrial genomes of the *Apis cerana*-Diannan and *Apis cerana*-Yun-Gui Plateau populations by using PacBio HiFi sequencing technology. The *A. cerana*-Diannan and *A. cerana*-Yun-Gui Plateau mitochondrial genome lengths were 16,214 and 16,304 bp, respectively. Both *A. cerana* mitochondrial genomes contained 13 protein-coding genes, 22 transfer RNAs, 2 ribosomal RNAs and an AT-rich region. The length of the AT-rich region of two *A. cerana* was different. Phylogenetic analyses showed that these two *A. cerana* populations belong to the same phylogenetic branch, but the genetic distance between DN and YG is further than that between YG and *A. cerana*-Aba and *A. cerana*-Central China. Our study shows that different geographical distributions of *A. cerana* show genetic diversity at the mitochondrial genome level.

## 1. Introduction

The honeybee *Apis cerana* plays a crucial role as a pollinator, contributing significantly to biodiversity and supporting ecological processes. Due to the complex and varied topography in China, *A. cerana* has evolved in diverse environments and is now widely distributed across most regions of the country [1]. In 2011, the National Animal Genetic Resources Committee summarized previous research and classified *A. cerana* into nine ecological groups, including the Changbaishan, North China, Central China, South China, Tibet, Aba, the Yun-Gui Plateau, Diannan and Hainan groups [2]. However, the *A. cerana* populations have declined in some regions due to climate change, human activities and bee diseases [3,4]. Thus, the study of genetic diversity is fundamental to the conservation and utilization of honeybee resources and is particularly crucial for *A. cerana* populations that are experiencing rapid declines. Furthermore, it is important to elucidate the mechanisms of *A. cerana* environmental adaptation.

Assessments of honeybee genetic diversity have included morphological comparisons and the identification of molecular markers [5]. Relying solely on morphological characteristics to differentiate *A. cerana* groups has limitations, whereas molecular markers are recognized as a more reliable and accurate method [6]. Molecular markers are inheritable DNA sequences that can be precisely identified and are used to map genetic variations, including those involving proteins [7]. At present, mitochondrial DNA (mtDNA) markers are among the most frequently used molecular marker techniques [8]. Due to their rapid rate of evolution, simple structural composition and abundant genetic information, mitochondria have become crucial tools for studying the evolutionary origins and genetic diversity of organisms [9]. *A. cerana* exhibits a consistent matrilineal mitochondrial pattern and is an important pollinating insect; thus, it is considered an ideal organism for studying genetic diversity via the mitochondrial genome. Research has consistently shown that the mitochondrial genome varies significantly across species, subspecies and geographic groups of honeybees, making it an effective tool for studying genetic diversity and evolution in these insects [10,11].

Improvements in sequencing technologies and bioinformatic approaches make it feasible to detect genomic variation and to reveal the genomic properties associated with the process of adapting to local environments [12]. Sequencing technology is widely used for analyzing both gene fragments and whole genomes to assess genetic diversity [13,14]. Next-generation sequencing (NGS) analysis of the evolution of the *A. mellifera* mitochondrial genome and haplotype analysis of the *COX3* gene have revealed genomic differences in *A. mellifera* at the subspecies level [15]. Sequencing of the complete mitochondrial genome and exons of the gene vitellogenin (VG) nuclear genome revealed genomic differences in the populations of *A. cerana* of the Far East of Russia, Korea and Japan at the subspecies level [16,17]. Third-generation sequencing (TGS) is currently dominated by the PacBio and Oxford Nanopore technologies. Compared with NGS, TGS provides longer read lengths, which has obvious advantages for specific applications and positions TGS as the predominant sequencing method today [18,19].

In this study, we collected two *A. cerana* groups from two different geographical locations. These populations correspond to the geographic populations *A. cerana*-Diannan (DN) and *A. cerana*-Yun-Gui Plateau (YG). The complete mitochondrial genomes of these two geographically distinct populations of *A. cerana* were assembled using PacBio HiFi sequencing technology. We identified genetic similarities and differences between the two *A. cerana* populations by comparing their mitochondrial genomes. This study will contribute to elucidating the genetic differentiation of *A. cerana* populations in various environments.

## 2. Materials and Methods

### 2.1. Sample Collection and DNA Sequencing

**Figure 1:**
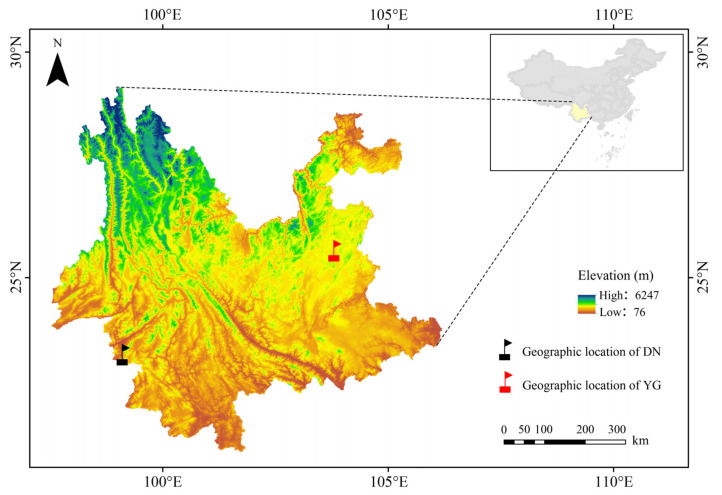
Sampling site information on DN and YG.

The DN samples were obtained from the Yunnan Cangyuan Experimental Station of the Institute of Apicultural Research, Chinese Academy of Agricultural Sciences, Cangyuan County, Yunnan Province (23°18′00″° N, 99°7′12″ E, 1251.4 m elevation), and the YG samples were obtained from an apiary located in Zhanyi District, Yunnan Province (25°36′00″ N, 103°49′12″ E, 1857.8 m elevation) (Figure 1). Fifteen drone pupae of DN and fifteen drone pupae of YG were sampled, placed in absolute ethanol and stored in a −20 °C freezer. Genomic DNA (gDNA) was extracted from one drone thorax per colony, following the Blood & Cell Culture DNA Kit (Qiagen Cat.13323, Hilden, German). DNA quality was assessed through three methods: NanoDrop spectrophotometry, gel electrophoresis and Qubit fluorometry. High-purity gDNA (≥10 μg, ≥100 ng/μL) was then prepared for library construction after purification with AMPure PB beads.

We constructed SMRTbell libraries using the SMRTbell Express Template Prep Kit 2.0 (PN 101-853-100, Pacific Biosciences, Menlo Park, CA, USA) following standard procedures. Briefly, DNA (10 μg) was assessed for fragment length (>40 Kb via pulsed-field electrophoresis), sheared to approximately 15 Kb and further processed for library construction, including end repair, adapter ligation and size selection via SageELF or BluePippin (Sage Science, Inc., Beverly, MA, USA). The final libraries were sequenced for 30 h using the Sequel II/IIe system (Pacific Biosciences, Menlo Park, CA, USA).

### 2.2. Mitochondrial Genome Assembly and Annotation

PacBio HiFi data for DN and YG were assembled using Hifiasm (0.19.5-r587) software [20,21]. The assembled genome was used to construct a local BLAST database. A reference *A. cerana* mitochondrial genome (GenBank: NC014295) was downloaded to search the local BLAST database. The MITOS Web Server (http://mitos.bioinf.uni-leipzig.de/result.py?name=DNmito_pilon&hash=S4tfplk8&no=0, accessed on 7 July 2023) [22] was used for preliminary annotation of the mitochondrial genome, and Chlorobox GeSeq (https://chlorobox.mpimp-golm.mpg.de/geseq.html, accessed on 7 July 2023) [23] was subsequently used to compare the annotated preliminary results with previously reported protein and RNA sequences for related species to verify the accuracy of the results and to correct them. A circular map of the mitochondrial genome was drawn based on the annotation results using the online tool Chloroplot v.0.2.4 (https://irscope.shinyapps.io/Chloroplot/, accessed on 12 July 2023) [24].

### 2.3. Mitochondrial Genome Sequence Analysis

BioEdit v.7.0.9.0 [25] was used to calculate the base composition and codon usage of the complete DN and YG mitochondrial genome sequence and the protein-coding genes (PCGs), tRNA genes, rRNA genes and AT-rich regions. Codon usage bias, represented by relative synonymous codon usage (RSCU), was calculated using codonW v.1.4.2 and was plotted into a bar graph using JSHYCloud (http://cloud.genepioneer.com:9929, accessed on 6 August 2023). An RSCU greater than 1 indicated a preference for certain amino acids. The AT and GC biases were calculated according to the following formulae: AT skew = (A − T)/(A + T) and GC skew = (G − C)/(G + C). MEGA v.11 [26] was used to compare the AT-rich region sequences of the two *A. cerana* groups with insertions or deletions.

To verify the AT-rich region in the two geographically distinct *A. cerana* populations, a sequence length analysis method was used. First, the BLASTN + 2.13.0 was used to screen mitochondrial genome sequences from the PacBio HiFi data for DN and YG. When the BLAST similarity to the *A. cerana* mitochondrial genome was greater than 80%, sequences were collected. Second, the left and right 400 bp lateral sequences of the AT-rich region were extracted, and a local database was constructed. Then, the -cx asm5 command of the Minimap2 [27] was used to align and collect sequences in which the length of the overlay region was more than 50 bp in AT-rich regions. The redundancy of duplicate sequences was addressed, and frequency distribution analysis was performed to determine the frequency distribution for different lengths of the mitochondrial AT-rich region.

### 2.4. Phylogenetic Analyses

The complete *A. cerana* mitochondrial genome was retrieved from the NCBI GenBank database. A total of 17 honeybee mitochondrial genomes were used in this study, including those of DN and YG, 13 published sequences and those of *A. mellifera sinisxinyuan* and *A. mellifera capensis* as outgroups (Table 1). Due to tRNA genes and rRNAs being highly conserved in base sequences and secondary structures, utilizing tRNAs and rRNAs in phylogenetic analyses was less accurate. Therefore, 13 PCGs were used to analyze the phylogenetic relationships of *A. cerana* groups from different geographic regions. The 13 PCG sequences were input into PhyloSuite v.1.2.3 [28,29] and aligned using MAFFT v.7.313 [30]. Then, the PCG sequences were concatenated. In the selection of evolutionary models, we used ModelFinder v.2.1.7 [31] based on the Bayesian information criterion (BIC) to determine the best model. The BIC not only considers the goodness of fit of the model but also penalizes the complexity of the model to avoid overfitting. The evolutionary tree was constructed using MrBayes v.3.2.6 [32] with a chain length set to 10 million generations, sampling every 1000 generations and discarding the first 25% as burn-in. Additionally, IQ tree v.1.6.8 [33] was used with bootstrap support values based on 1000 replicates.

## 3. Results

### 3.1. Mitogenome Organization and Base Composition

**Figure 2:**
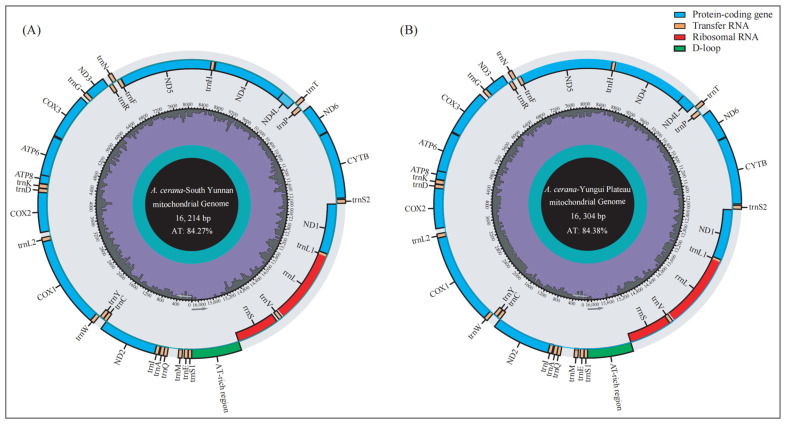
Graphical maps of the mitochondrial genomes of DN (**A**) and YG (**B**). The inside circles show the G + C content.

We assembled the complete DN (GenBank: PP175371) and YG (GenBank: PP175370) mitochondrial genomes (Figure 2). The length of the complete mitochondrial genome for DN was 16,214 bp, with an AT content of 84.27%, while that for YG was 16,304 bp, with an AT content of 84.38%. Both mitochondrial genomes were annotated with 37 genes, including 13 protein-coding genes (PCGs), 22 tRNA genes, 2 rRNA genes and an AT-rich region located between *srRNA* and trnS1 (Table 2). Among the 37 genes, 23 were located on the J chain (majority strand), and the remaining 14 were located on the N chain (minority strand) (Figure 2 and Table 2). There was no difference in the number of initiation or termination codons between the two *A. cerana* groups. The initiation codons of all the PCGs were standard ATN (T/C/G), and the termination codons were T(AA). The lengths of the DN and YG intergenic regions ranged from 0 to 230 bp and 0 to 231 bp, respectively. The *ATP8* and *ATP6* genes share 19 nucleotides.

### 3.2. Protein-Coding Genes

The total length of the 13 PCGs in both DN and YG was 11,048 bp, accounting for 68.14% and 67.76%, respectively, of the entire mitochondrial genome (Table 3). Except for the A + T content of *COX1* and *COX2* in DN and YG, the A + T content of the other PCGs was greater than 80%, indicating an AT preference. According to the AT-skew and GC-skew results, most of the PCGs were biased toward T, while all PCGs showed a bias toward C.

**Figure 3:**
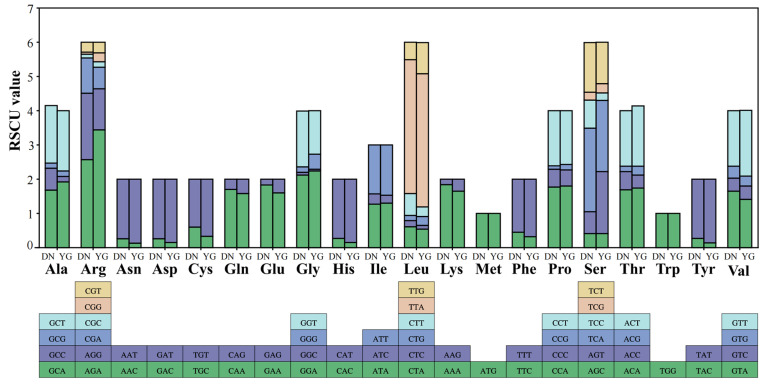
Codon bias statistics for 13 protein-coding genes of DN and YG.

We analyzed the frequency and RSCU values for 13 PCGs in the DN and YG mitochondrial genomes. The RSCU values for the 13 PCGs were greater than 1, except for those of the two amino acids Met and Trp, which were encoded by only one codon (Figure 3), indicating that all codons were biased. The TTA codon had the highest RSCU value in both *A. cerana* groups (3.91 in DN and 3.89 in YG), followed by the AGA codon (2.57 in DN and 3.44 in YG). NNA-type codons accounted for 42.82% and 42.33% of the total number of codons, indicating that codons with an A at the third position were the most frequent.

### 3.3. Transfer RNAs and Ribosomal RNAs

The typical set of 22 tRNAs was scattered throughout each circular, double-stranded DNA molecule, ranging from 60 bp for trnS1 to 78 bp for trnP (Table 2). The total tRNA lengths in DN and YG were 1486 bp and 1487 bp, respectively (Table 4). The tRNA A + T% was 87.28% for DN and 87.35% for YG. Both large ribosomal RNA (*lrRNA*) and small ribosomal RNA (*srRNA*) were located on the N chain, and the lengths of the *lrRNA*s and *srRNA*s in the two *A. cerana* groups were 1322 bp and 773 bp, respectively, and the difference in the length of the tRNAs was 1 bp. Therefore, there was no significant difference in the length of tRNAs and rRNAs between DN and YG. According to the AT-skew and GC-skew results, the T and C contents in rRNA and tRNA were greater.

### 3.4. AT-Rich Region

The AT-rich region in DN and YG was situated on the N strand between the *srRNA* and trnS1. The A + T% in the two *A. cerana* groups accounted for 97.43% and 97.45%, respectively, in the AT-rich region. We initially identified 85 bp fragment insertions and deletions in the AT-rich region through sequence comparisons of DN and YG (Table 3). The frequency distribution statistics on the fragment length showed that the length distribution of the AT-rich region in most DN fragments was approximately 900 bp, while the length distribution of most YG fragments was approximately 1000 bp, with a difference of approximately 100 bp. The frequency distribution plot for the length of the AT region confirmed that there was a difference in the length for the two *A. cerana* groups.

**Figure 4:**
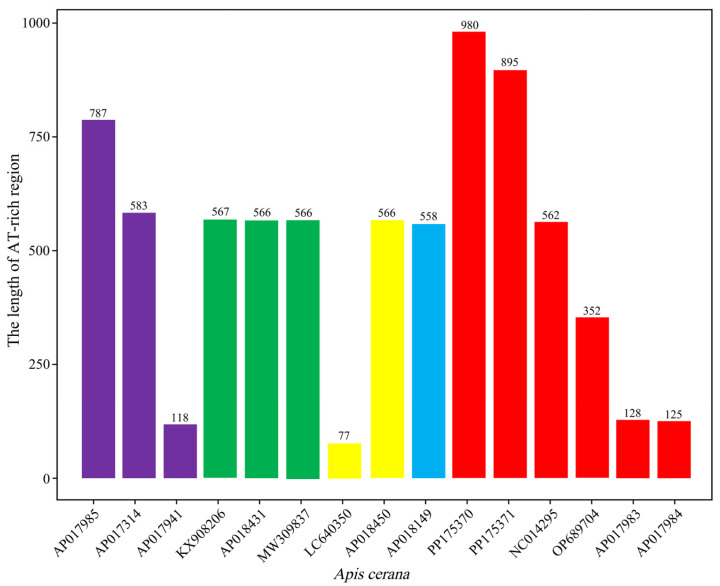
The length statistics of the AT-rich region of *A. cerana*. Purple represents *A. cerana* from Japan; green represents *A. cerana* from Korea; yellow represents *A. cerana* from Russia; blue represents *A. cerana* from Borneo; and red represents *A. cerana* from China.

According to the statistics of AT-rich region length of *A. cerana* from different geographical locations (Figure 4), there were differences in the length of AT-rich region of *A. cerana* from different regions of Japan, Russia and China, with the largest difference of 710 bp in length in Japan, 489 bp in length in Russia and 855 bp in length in China. The AT-rich region from South Korea was similar in length.

### 3.5. Phylogenetic Analysis

**Figure 5:**
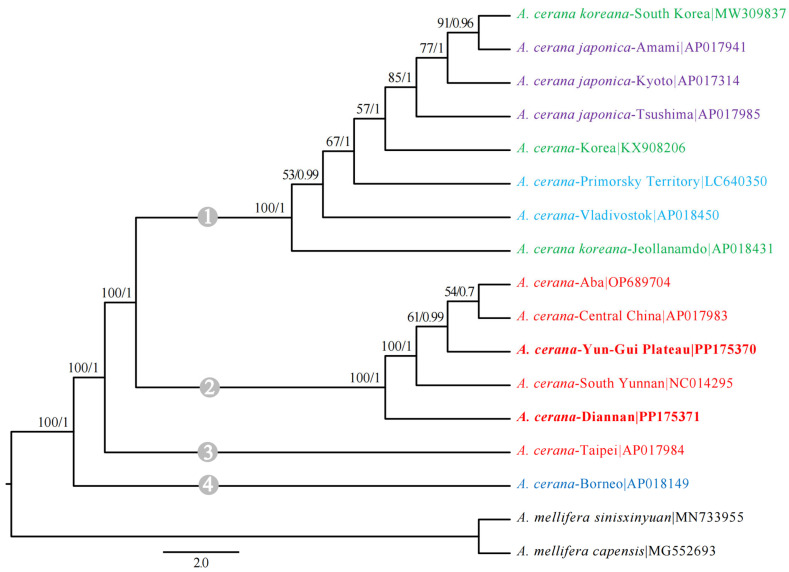
13 PCGs were used to analyze the phylogenetic relationships of *A. cerana*, *A. mellifera sinisxinyuan* and *A. mellifera capensis* as outgroups. The numbers shown at each branch point represent the support values for the nodes: the first number indicates the bootstrap support value derived from maximum likelihood analysis, while the second number represents the posterior probability obtained from Bayesian inference analysis. The same color *A. cerana* comes from the same country.

The results for the evolutionary tree showed that *A. cerana* was divided into four branches, one from Taiwan and one from Borneo, with high node support. *A. cerana* from Korea, Japan and Russia formed a single colony, while those from mainland China formed an additional colony (Figure 5). DN and other *A. cerana* in mainland China represent sister groups. The genetic distance of YG was closer to that of *A. cerana*-Aba and *A. cerana*-Central China. The geographical distribution distance between DN and YG was close, but the genetic distance between the two groups of *A. cerana* was farther than that between YG and *A. cerana*-Aba and *A. cerana*-Central China. The results for the evolutionary tree confirmed that there was a difference between DN and YG.

## 4. Discussion

In light of the destruction of *A. cerana* populations, it is vital to discover effective genetic markers to distinguish adjacent populations. Mitochondrial DNA is inherited maternally in bees. A single bee can therefore be used to represent an entire colony. Here, we compared the DN and YG mitochondrial genomes to assess the differences among major *A. cerana* groups. An insertion or deletion was first found in the AT region from different geographic populations.

Advances in sequencing technologies and TGS have enabled researchers to explore the complex structure of these genomes more accurately [38]. In this study, we utilized PacBio HiFi sequencing to assemble the complete mitochondrial genomes of two *A. cerana* groups, DN and YG. This approach provides an opportunity to explore the genetic diversity of *A. cerana* in different habitats. The use of HiFi sequencing proved particularly advantageous in addressing challenges associated with the AT-rich region, a non-coding segment characterized by high AT content, low complexity, GC bias, low coverage and issues with homopolymer runs. These challenges have traditionally increased the complexity of genome assembly, especially with short-read sequencing technologies. By generating high-quality long-read data, HiFi sequencing significantly enhanced coverage and assembly accuracy in the AT-rich region, ensuring the reliability of our results and demonstrating the effectiveness of this technology in resolving such regions. According to our results, the full lengths of the DN and YG mitochondrial genomes were 16,214 bp and 16,304 bp, respectively. This finding aligns with previous reports indicating that animal mitochondria typically range between 10 and 20 kb in length [39]. Both mitochondrial genomes in the present study contained 37 common genes (13 PCGs, 22 tRNAs and 2 rRNAs) and exhibited similar AT preferences and codon usage frequencies, consistent with the findings for *A. cerana japonica* [35]. Mitochondrial genome rearrangement has been previously reported in lepidoptera, but this study of the two *A. cerana* groups showed no rearrangement, which is considered to indicate the ground pattern of insect mitochondrial genomes [40,41]. Whether sequences tend to be A-encoded or T-encoded depends on the role they play. Strand asymmetry, also known as strand compositional bias, is typically indicated by the AT and the GC skews [42]. The two *A. cerana* groups in the present study exhibited a reversal of strand asymmetry on the entire majority strand, possibly due to the inversion of the replication origin situated in the control region [43]. rRNAs are commonly used to classify species [44]. A comparison of the tRNA and rRNA sequences from DN and YG revealed that both regions were approximately the same length, with a minor difference in A + T content. Previous studies have shown that in insect mitochondrial genomes, AT-rich regions typically regulate gene replication and transcription. The lengths of these sequences vary significantly among insects and even within genera [45,46]. Our results support this concept.

We conducted codon preference analysis on 13 PCGs to identify variations, with the aim of identifying differences in differentiation between DN and YG at the gene level by examining codon frequencies. The eukaryotic genome contains 64 codons that encode 20 different amino acids and 3 stop codons [47]. All amino acids, except Met and Trp, are encoded by two to six synonymous codons [48]. The preference for the utilization of these synonymous codons is determined by several factors, including the abundance of tRNAs, the mutational bias of the gene chain, the gene expression level, the gene length and the gene expression level [49]. The results for the RSCU analysis (Figure 3) revealed variations in codon usage frequency between the two *A. cerana* groups, likely attributed to varying levels of differentiation. We found that *A. cerana* exhibits a preference for synonymous codons ending in A or T across various amino acids. This trend is consistent with findings in other insect species. For example, a study on *Bactericera cockerelli* reported a similar codon usage pattern, where codons ending in U and A were most frequently used, attributed to the high A + T content in its mitochondrial genome [44]. The potential reasons for RSCU bias have been examined in the genomes of various living organisms, such as *A. laboriosa*, scuticociliates, sesame and astrovirus [10,50,51,52]. Geographical distance and geographical location were correlated with the genetic differentiation of *A. cerana.* This is attributed to the distinct living environments of DN and YG. The sampling sites for DN and YG in this study were, respectively, located in Cangyuan County in the southern part and Zhan Yi District in the eastern part of Yunnan Province. These two locations exhibit distinct environmental climates: Cangyuan County features a subtropical monsoon climate, while Zhan Yi District has a subtropical humid monsoon climate [53].

The AT-rich region is well known for its ability to initiate replication in both vertebrates and invertebrates, and a reduced G + C content is one of the most prominent features of this region [39]. The mitochondrial genome insertion and deletion regions were analyzed in DN and YG. Our findings revealed that the AT-rich region had the highest degree of variation in terms of nucleic acid sequence and length. There were not only interspecific differences in copy number but also variations among individuals within the same species. The reliability of copy number variations for species identification has been demonstrated in previous studies [54,55]. The consistency in the phylogenetic topology of most clades indicates that AT-rich regions generally evolve under similar evolutionary pressures as mitochondrial PCGs or mitogenomes [56]. We speculate that the insertion and deletion of sequences in the AT-rich region of *A. cerana* from the DN and YG provide evidence for subspecies classification. This region is highly variable and lacks pairwise sites that can be used for phylogenetic analysis, making sequence alignment difficult. The statistical comparison of AT-rich region length (Figure 4) showed that the length of AT-rich region in different environments in the same country was also different, which might be related to different living environments. The significant variability observed in the Chinese, Russian and Japanese populations, in particular, suggests that honeybee populations in these regions may have experienced stronger ecological selection pressures, leading to genetic differentiation. Previous studies on the AT-rich region have shown that the method for species classification on the basis of this region is feasible [46]. We identified several elements typically found in the AT-rich regions of most insects, including the TATA motif, which likely plays a role in initiating genome replication. Although the AT-rich region is highly variable, its downstream conserved regions can be partially aligned to reflect phylogenetic positions altered by evolutionary forces. The variations observed in the AT-rich region may have functional significance, potentially influencing mitochondrial DNA replication and transcriptional activity. As this region often contains replication origins, such variations may affect replication efficiency or regulatory function [57]. In our study, we found structural and sequence differences in the AT-rich region between the two populations, which could alter secondary DNA structures and affect the stability of replication and transcription.

The mitochondrial phylogeny suggests that the current population structure of *A. cerana* on the Chinese mainland is the result of multiple differentiations, potentially shaped by complex historical and environmental factors. The phylogenetic tree we constructed indicates that *A. cerana* strains from Taiwan and Borneo are distinct, with YG being a sister group to *A. cerana*-Aba and *A. cerana*-Central China. Despite these phylogenetic differentiations, we observed no significant correlation between genetic diversification and geographical distance. This pattern suggests that historical events, such as glacial cycles or population isolation during climatic shifts, may have played a role in shaping the current genetic structure. Previous studies on honeybees across various regions, including analyses of morphological differences between DN and YG, indicate that the Tropic of Cancer serves as a geographic boundary [58], further supporting the idea that physical barriers rather than geographic distance alone are a major driver of population divergence in *A. cerana* [14]. In contrast, environmental factors, particularly altitude, have been shown to influence genetic differentiation in honeybee populations [59,60], potentially through adaptive mechanisms, such as cold resistance and metabolic efficiency at high altitudes [61]. Genomic incompatibilities, such as structural variations and local adaptations, could further reinforce these patterns, highlighting the role of specific environmental pressures. Additionally, we hypothesize that the genetic uniqueness and basal phylogenetic position of the Taiwan island subspecies could be explained by a relatively long divergence time due to marine isolation, which has limited gene flow with mainland populations. Alternatively, accelerated urbanization and the introduction of alien honeybee species [62,63] may have influenced the genetic structure, particularly by promoting hybridization or population bottlenecks. Further comparison with historical samples and additional studies focusing on environmental and ecological variables will be necessary to disentangle the historical and recent anthropogenic impacts on genetic distance patterns in *A. cerana*.

In contrast to previous studies that provided only limited information about *A. cerana* and a few studies that reported comparative analyses of *A. cerana* at the genetic level, the mitochondrial genome-wide analysis of *A. cerana* in our study offers a more detailed genetic structure of honeybees. Comparative genome-wide analyses of DN and YG revealed intraspecific genetic evolutionary differences among the various *A. cerana* groups. Moreover, the differences and uniqueness of the DN and YG mitochondrial genomes provide insights into *A. cerana* species differentiation. This study lays an important foundation for the husbandry and conservation of *A. cerana* in various regions. In future, various *A. cerana* taxa can be distinguished based on structural and genetic evolutionary variances. Targeted conservation measures could include protecting the natural habitats of different *A. cerana* groups to preserve their unique genetic traits, establishing a genetic diversity conservation bank and prioritizing populations with high genetic diversity. Gene flow between populations in neighboring regions could also be promoted where appropriate to enhance adaptability and prevent inbreeding. Such targeted species protection measures can then be implemented according to the distinct groups to increase *A. cerana* population sizes and preserve its genetic diversity. This study also has several shortcomings. Due to the limited number of *A. cerana* samples collected, morphological analysis could not be performed in this study. More in-depth studies on species differentiation can be conducted in the future by combining morphological and genomic studies. For systemic evolution, the maximum likelihood analysis and Bayesian inference analysis have certain limitations, such as computational demands and sensitivity to model assumptions. Future studies could consider complementary approaches, such as coalescent-based species tree methods or phylogenetic network analyses, to provide a more comprehensive view of population structure.

## 5. Conclusions

In conclusion, we utilized PacBio HiFi sequencing technology to assemble and annotate the DN and YG mitochondrial genomes, resulting in the assembly of two high-quality organelle genomes. By comparing the mitochondrial genomes, we found that *A. cerana* exhibits genetic diversity at the mitochondrial genomic level in various environments. The difference in the insertion or deletion of long fragments in AT-rich regions between the *A. cerana* groups serves as the basis for species differentiation. The results for the phylogenetic tree indicated differences between the two closely related *A. cerana* groups, DN and YG. This study expands the classification of *A. cerana*. It is hoped that more specimens can be collected to conduct a more comprehensive and systematic study of honeybees. The morphological data and gene fragments can be utilized for combined analyses in future.

## Figures and Tables

**Table 1:** Information on the sequences in the phylogenetic tree.

Number	Species	GenBank Accession Number	Sampling Locality	Reference
1	*Apis cerana japonica*-Tsushima	AP017985.1	Japan	[34]
2	*Apis cerana japonica*-Kyoto	AP017314.1	Japan	[35]
3	*Apis cerana japonica*-Amami	AP017941.1	Japan	[34]
4	*Apis cerana*-Primorsky Territory	LC640350.1	Russia	Directly Submitted
5	*Apis cerana*-Korea	KX908206.1	Korea	Directly Submitted
6	*Apis cerana koreana*-Jeollanamdo	AP018431.1	Korea	Directly Submitted
7	*Apis cerana koreana*-South Korea	MW309837.1	Korea	Directly Submitted
8	*Apis cerana*-Vladivostok	AP018450.1	Russia	[17]
9	*Apis cerana*-Borneo	AP018149.1	Borneo	[36]
10	*Apis cerana*-South Yunnan	NC014295.1	China	[37]
11	*Apis cerana*-Aba	OP689704.1	China	Directly Submitted
12	*Apis cerana*-Central China	AP017983.2	China	[34]
13	*Apis cerana*-Taipei	AP017984.2	China	[34]
14	*Apis mellifera sinisxinyuan* *	MN733955.1	China	Directly Submitted
15	*Apis mellifera capensis* *	MG552693.1	South Africa	[15]

Note: “Directly Submitted” indicates that the sequence was submitted directly to the GenBank database by the research team and has not been published in a formal journal. The asterisk represents the outgroup.

**Table 2:** Annotations of the mitogenomes of two complete mitochondrial genomes (*A. cerana*-Diannan/*A. cerana*-Yun-Gui Plateau).

Gene	Start Position	End Position	Length/bp	Intergenic Nucleotides/bp	Initiation Codons	Termination Codons	Direction
trnS1(Ser)	1/1	60/60	60/60				J/J
trnE(Glu)	64/64	129/129	66/66	3/3			J/J
trnM(Met)	164/164	229/229	66/66	34/34			J/J
trnQ(Gln)	461/461	522/522	62/62	230/231			J/J
trnA(Ala)	523/523	588/588	66/66	0/0			J/J
trnI(Ile)	607/607	672/672	66/66	18/18			J/J
ND2	673/673	1668/1668	996/996	0/0	ATT/ATT	TAA/TAA	J/J
trnC(Cys)	1668/1668	1733/1733	66/66	−1/−1			N/N
trnY(Tyr)	1739/1739	1807/1807	69/69	5/5			N/N
trnW(Trp)	1824/1824	1892/1892	69/69	16/16			J/J
COX1	1893/1893	3453/3453	1561/1561	0/0	ATT/ATT	T(AA)/T(AA)	J/J
trnL2(Leu)	3454/3454	3523/3523	70/70	0/0			J/J
COX2	3613/3613	4291/4291	679/679	89/89	ATT/ATT	T(AA)/T(AA)	J/J
trnD(Asp)	4292/4292	4359/4359	68/68	0/0			J/J
trnK(Lys)	4366/4366	4437/4437	72/72	6/6			J/J
ATP8	4444/4444	4605/4605	162/162	6/6	ATC/ATC	TAA/TAA	J/J
ATP6	4587/4587	5264/5264	678/678	−19/−19	ATG/ATG	TAA/TAA	J/J
COX3	5282/5282	6061/6061	780/780	17/17	ATG/ATG	TAA/TAA	J/J
trnG(Gly)	6125/6132	6191/6198	67/67	63/70			J/J
ND3	6192/6199	6545/6552	354/354	0/0	ATT/ATT	TAA/TAA	J/J
trnR(Arg)	6566/6572	6631/6637	66/66	20/19			N/N
trnN(Asn)	6651/6657	6718/6724	68/68	19/19			J/J
trnF(Phe)	6737/6743	6807/6813	71/71	18/18			N/N
ND5	6814/6820	8481/8487	1668/1668	6/6	ATT/ATT	TAA/TAA	N/N
trnH(His)	8483/8489	8548/8554	66/66	1/1			N/N
ND4	8566/8572	9894/9900	1329/1329	17/17	ATT/ATT	TAA/TAA	N/N
ND4L	9895/9901	10,158/10,164	264/264	0/0	ATT/ATT	TAA/TAA	N/N
trnT(Thr)	10,182/10,188	10,248/10,254	67/67	23/23			J/J
trnP(Pro)	10,264/10,270	10,341/10,347	78/78	15/15			N/N
ND6	10,392/10,398	10,904/10,910	513/513	50/50	ATT/ATT	TAA/TAA	J/J
CYTB	10,917/10,923	12,065/12,071	1149/1149	12/12	ATG/ATG	TAA/TAA	J/J
trnS2(Ser)	12,089/12,095	12,155/12,161	67/67	23/23			J/J
ND1	12,168/12,174	13,082/13,088	915/915	12/12	ATT/ATT	TAA/TAA	N/N
trnL1(Leu)	13,083/13,089	13,151/13,157	69/69	0/0			N/N
large subunitr RNA(lrRNA)	13,152/13,158	14,451/14,456	1322/1322	0/0			N/N
trnV(Val)	14,452/14,457	14,546/14,552	67/68	0/0			N/N
small subunit rRNA(srRNA)	14,547/14,553	15,319/15,324	773/773	0/0			N/N
AT-rich region	15,320/15,325	16,214/16,304	895/980	0/0			J/J

**Table 3:** Codon usage of 13 protein-coding genes and AT-rich regions.

Region	Length (bp)		A%		T%		C%		G%		A + T%		AT-Skew		GC-Skew	
	DN	YG	DN	YG	DN	YG	DN	YG	DN	YG	DN	YG	DN	YG	DN	YG
ND2	996	996	39.26	39.36	46.89	47.19	8.63	8.23	5.22	5.22	86.15	86.55	−0.09	−0.09	−0.25	−0.22
COX1	1561	1561	34.66	34.66	41.38	41.19	12.88	13.07	11.08	11.08	76.04	75.85	−0.09	−0.09	−0.05	−0.08
COX2	679	679	37.85	38.44	40.65	40.35	12.37	12.67	9.13	8.54	78.5	78.79	−0.04	−0.02	−0.15	−0.19
ATP8	162	162	47.53	47.53	38.89	39.51	9.26	8.64	4.32	4.32	86.42	87.04	0.1	0.09	−0.36	−0.33
ATP6	678	678	36.73	36.58	47.49	47.49	10.18	10.33	5.6	5.6	84.22	84.07	−0.13	−0.13	−0.29	−0.3
COX3	780	780	36.41	35.9	44.23	44.61	10.64	10.64	8.72	8.85	80.64	80.51	−0.1	−0.11	−0.1	−0.09
ND3	354	354	37.57	37.57	48.02	47.74	9.61	9.6	4.8	5.09	85.59	85.31	−0.12	−0.12	−0.33	−0.31
ND5	1668	1668	46.94	47.06	37.95	37.89	9.53	9.47	5.58	5.58	84.89	84.95	0.11	0.11	−0.26	−0.26
ND4	1329	1329	49.13	49.06	35.89	35.97	9.49	9.41	5.49	5.56	85.02	85.03	0.16	0.15	−0.27	−0.26
ND4L	264	264	52.65	52.65	34.47	34.47	9.47	9.47	3.41	3.41	87.12	87.12	0.21	0.21	−0.47	−0.47
ND6	513	513	43.08	43.08	43.08	43.27	8.58	8.38	5.26	5.27	86.16	86.35	0	0	−0.24	−0.23
CYTB	1149	1149	36.73	36.73	44.21	44.21	10.53	10.53	8.53	8.53	80.94	80.94	−0.09	−0.09	−0.1	−0.1
ND1	915	915	47.98	47.98	35.52	35.52	10.93	10.93	5.57	5.57	83.5	83.5	0.15	0.15	−0.32	−0.32
AT-rich region	895	980	47.71	47.45	49.72	50	1.56	1.33	1.01	1.22	97.43	97.45	−0.02	−0.03	−0.21	−0.04

**Table 4:** Codon usage of rRNA genes and tRNA genes.

Region	Length (bp)		A%		T%		C%		G%		A + T%		AT-Skew		GC-Skew	
	DN	YG	DN	YG	DN	YG	DN	YG	DN	YG	DN	YG	DN	YG	DN	YG
lrRNA	1322	1322	42.46	42.57	40.47	40.42	11.15	11.16	5.92	5.85	82.92	82.99	0.02	0.03	−0.31	−0.31
srRNA	773	773	37.52	37.56	43.85	43.91	12.55	12.44	6.08	6.09	81.37	81.47	−0.08	−0.08	−0.35	−0.34
rRNA	2095	2095	40.62	40.70	41.73	41.72	11.67	11.64	5.98	5.94	82.35	82.42	−0.01	−0.01	−0.32	−0.32
tRNA	1486	1487	42.33	42.43	44.95	44.92	7.40	7.40	5.32	5.25	87.28	87.35	−0.03	−0.03	−0.16	−0.17

## Data Availability

The datasets generated and analyzed in the current study are available in the National Center for Biotechnology Information (NCBI) repository under the accession numbers PP175370.1 and PP175371.1.
